# Heat stress and bleaching in corals: a bioenergetic model

**DOI:** 10.1007/s00338-024-02561-1

**Published:** 2024-10-30

**Authors:** Ferdinand Pfab, A. Raine Detmer, Holly V. Moeller, Roger M. Nisbet, Hollie M. Putnam, Ross Cunning

**Affiliations:** 1 Department of Ecology, Evolution and Marine Biology, University of California, Santa Barbara, CA USA; 2 Department of Biological Sciences, University of Rhode Island, Kingston, RI USA; 3 Conservation Research Department, John G. Shedd Aquarium, Chicago, IL USA

**Keywords:** Coral bleaching, Heat stress, Bioenergetic model, Simulation, DEB

## Abstract

The coral-dinoflagellate endosymbiosis is based on nutrient exchanges that impact holobiont energetics. Of particular concern is the breakdown or dysbiosis of this partnership that is seen in response to elevated temperatures, where loss of symbionts through coral bleaching can lead to starvation and mortality. Here we extend a dynamic bioenergetic model of coral symbioses to explore the mechanisms by which temperature impacts various processes in the symbiosis and to enable simulational analysis of thermal bleaching. Our model tests the effects of two distinct mechanisms for how increased temperature impacts the symbiosis: 1) accelerated metabolic rates due to thermodynamics and 2) damage to the photosynthetic machinery of the symbiont caused by heat stress. Model simulations show that the model can capture key biological responses to different levels of increased temperatures. Moderately increased temperatures increase metabolic rates and slightly decrease photosynthesis. The slightly decreased photosynthesis rates cause the host to receive less carbon and share more nitrogen with the symbiont. This results in temporarily increased symbiont growth and a higher symbiont/host ratio. In contrast, higher temperatures cause a breakdown of the symbiosis due to escalating feedback that involves further reduction in photosynthesis and insufficient energy supply for $\hbox {CO}_2$ concentration by the host. This leads to the accumulation of excess light energy and the generation of reactive oxygen species, eventually triggering symbiont expulsion and coral bleaching. Importantly, bleaching does not result from accelerated metabolic rates alone; it only occurs as a result of the photodamage mechanism due to its effect on nutrient cycling. Both higher light intensities and higher levels of DIN render corals more susceptible to heat stress. Conversely, heterotrophic feeding can increase the maximal temperature that can be tolerated by the coral. Collectively these results show that a bioenergetics model can capture many observed patterns of heat stress in corals, such as higher metabolic rates and higher symbiont/host ratios at moderately increased temperatures and symbiont expulsion at strongly increased temperatures.

**Supplementary Information:**

The online version contains supplementary material available at 10.1007/s00338-024-02561-1.

## Introduction

The main driver of the global decline of coral reefs is thought to be increased water temperatures due to climate change (Hoegh-Guldberg 1999). Heat stress is known to have multiple effects on corals, including damage to the photosynthetic machinery of the endosymbiotic algae (Family Symbiodiniaceae). This damage is associated with decreased photosynthesis rates, increased production of reactive oxygen species (ROS), and ultimately the expulsion of symbionts—which is observed as coral bleaching (Lesser 1997; Weis et al 2008). Other symptoms of heat stress include decreased translocation of carbohydrates from the symbiont to the host (Allen-Waller and Barott 2023), increased nitrogen available to the symbiont (Rädecker et al 2021), and (at moderately increased temperatures) increased cell division rates of the symbionts (Strychar et al 2004), as well as generally increased metabolic rates (Al-Horani 2005; Rädecker et al 2021). Some studies further suggest that $\hbox {CO}_2$ dynamics play an important role in photodamage. Reduced translocation of photosynthates during heat stress can impair the host’s carbon concentration mechanisms (CCMs), which then delivers less $\hbox {CO}_2$ to the symbiont and completes a vicious cycle by further decreasing the photosynthesis rate (Wooldridge 2009).

Much research has been conducted at various points of heat-induced coral bleaching (Cziesielski et al 2019; Helgoe et al 2024). However, less is known about where the cascade of bleaching events starts, and how the different steps interact (Gardner et al 2017a). To approach this gap from a modeling perspective, we here extend a bioenergetic model for the symbiosis between corals and their algal symbionts by Cunning et al (2017). This model accounts for light stress to the photosynthetic machinery, where exposure to excess light leads to ROS formation. In turn, ROS further decrease the photosynthesis rate (i.e., photoinhibition) and eventually trigger symbiont expulsion (i.e., bleaching). This model is part of a larger family of bioenergetic models for corals that are inspired by Dynamic Energy Budget (DEB) theory. The family of coral models includes predecessors that offer different levels of detail for photosynthesis and light stress (Muller et al 2009; Eynaud et al 2011) as well as to exemplify general modeling approaches (Pfab et al 2022), to investigate the effect of fish populations on coral colonies (Detmer et al 2022), and to investigate the role of multiple symbionts in corals (Brown et al 2022).

Coral bioenergetic modeling has successfully captured the nutrient cycling between the corals and their symbionts, as well as the effects of light intensity and nutrient abundance (Muller et al 2009; Eynaud et al 2011; Cunning et al 2017). It has generated critical knowledge on the connections of the different energy and nutrient fluxes in the system and identified hypotheses on the conditions under which the symbiosis breaks down because the system is pushed out of its equilibrium. However bioenergetic coral modeling has to date some limitations. These include the absence of temperature effects, which are critical to all enzymatic processes, and exacerbation of photodamage with increasing temperature (Lesser 2011). In particular, as thermal stress increases coral bleaching, it is essential to examine thermal impacts on a variety of critical aspects of coral-Symbiodiniaceae biology, including symbiont and host biomass growth and turnover rates, host feeding and nutrient uptake rates, and symbiont photosynthesis.

Here, we extend the bioenergetic model by Cunning et al (2017) by adding two primary temperature effects: 1) an acceleration of general metabolism and photosynthesis due to thermodynamic effects on enzymatic rate processes, and 2) damage to the photosynthetic machinery at very high temperatures.

For simplicity and generality, the model is not specific for a particular pathway for how heat stress initially causes damage (reviewed by Helgoe et al (2024)). The model aims at exploring qualitative behavior of the system. It is not specific for any particular coral and symbiont species, but rather aims at exploring qualitative behavior of a generalized coral holobiont.

We first analyze model simulations with both of the primary temperature effects together (metabolic acceleration and damage to the photosynthetic machinery) and observe how the effects of heat stress cascade through the modeled system. We then analyze simulations with each primary temperature effect separately to explore what consequences each of the effects has by itself. To expand the model analysis, we test how other environmental factors (variation in light, nutrients, and heterotrophy) affect the responses to temperature stress and the potential efficacy of interventions to manipulate these factors in mitigating coral bleaching.

The paper is structured as follows: section “Model and methods” offers an overview of the modeling and simulation approach, and section “Results and discussion” interprets the results in the context of the current understanding of coral bleaching. Specifically, section “Effects of changes in temperature on coral symbiosis” discusses the two primary temperature effects (damage to the photosynthetic machinery, and general metabolic acceleration), section “Interaction of environmental factors” discusses how temperature interacts with other environmental factors, section “Additional considerations and future directions” discusses biological underpinning of the model assumptions and model limitations, and section “Conclusion” summarizes the main findings. Appendix A and Appendix B describe the model details and the equations.

**Fig. 1:**
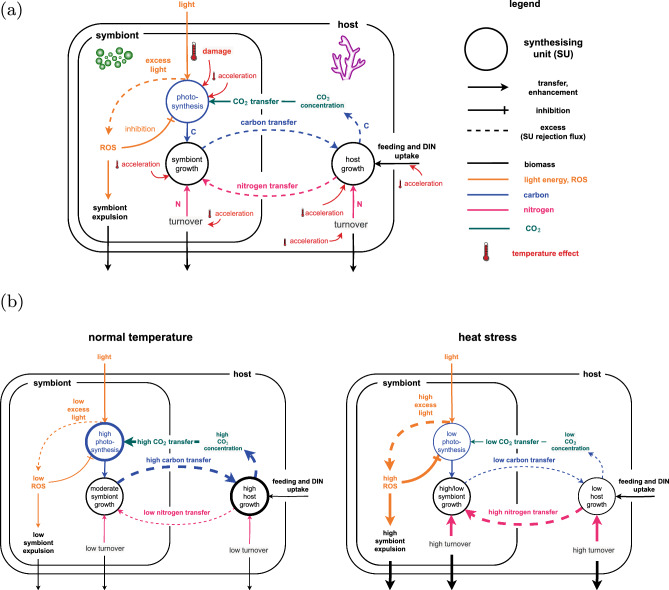
Model diagram. **1a** Overview. The model describes the effect of temperature on a coral host and its algae symbiont. Increased temperature is assumed to accelerate metabolic processes and damage the photosynthetic machinery of the symbiont. **1b** Primary and secondary effects of heat stress. Normal temperature: high photosynthesis, high carbon sharing by symbiont, low nitrogen sharing by host, moderate symbiont growth rate. The symbiosis is functional and the holobiont grows. Heat stress: acceleration of metabolic processes, damage to the photosynthetic machinery, and disruption of nutrient cycle. The symbiont shares less carbon and the host shares more nitrogen. At moderate heat intensities, the symbiont can have increased growth rates due to the additional nitrogen it receives from the host. At higher heat intensities, photosynthesis is reduced severely and the symbiosis breaks down rapidly. The photosystem is not capable of effectively transporting the captured excitation energy and produces large amounts of reactive oxygen species (ROS). The ROS eventually trigger symbiont expulsion and coral bleaching. These effects are further exacerbated through an escalating feedback in which the carbon concentration mechanisms (CCMs) of the host do not receive enough energy to fuel photosynthesis—which further increases the production of ROS and aggravates the impact of heat stress. A more detailed representation of the model system is shown in Fig. 7

## Model and methods

### Model description

**Fig. 2:**
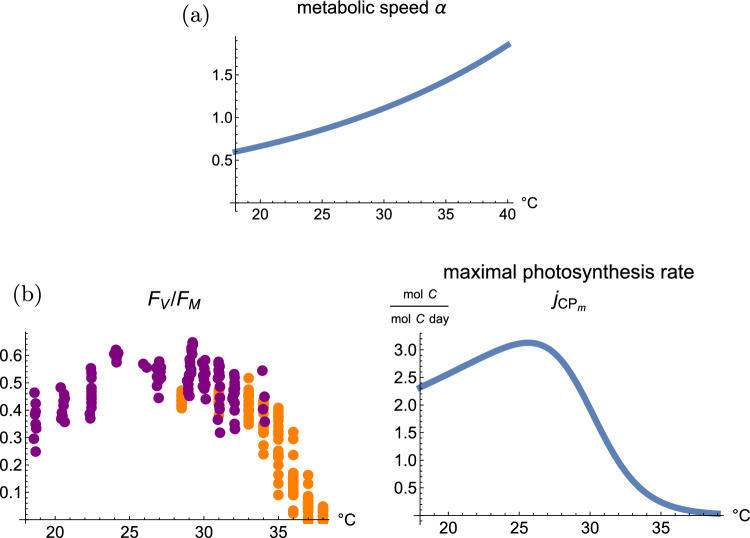
Direct effects of temperature in the model. 2a: The effect of temperature on metabolic speed. This function is a multiplier for six rates in the model: maximal symbiont growth rate, symbiont biomass turnover, maximal host growth rate, host biomass turnover, maximal host feeding rate, and maximal host DIN uptake. 2b: The effect of temperature on the photosynthetic machinery of endosymbiotic algae. Left: observed maximum quantum yield $F_v/F_m$ of corals at different temperatures from (Bellworthy and Fine 2021) on different coral species in the Red Sea (purple points to the left) and data from (Cunning 2024) on *Acropora pulchra* in Mo’orea, French Polynesia (orange points to the right). Right: maximal photosynthesis rate of symbionts in our model. The curve is composed of an increasing factor that corresponds to the general metabolic acceleration in the model (with the same rate as the other processes), and a steeper decreasing factor that accounts for heat-related damage to the photosynthetic machinery. The shape of the curve is originally fitted to the $F_v/F_m$ data on the left side by minimizing the sum of the square residuals. The maximum of the curve is set to be equal to the reference value in the original model by Cunning et al (2017). After fitting, we reduced the parameter for heat tolerance by $4^\circ \text {C}$ to achieve a more typical high-temperature bleaching threshold, because the data from Bellworthy and Fine (2021) were collected on particularly heat-adapted corals, and the data from Cunning (2024) were collected only after a short period of heat stress (7 h), presumably before the full damage level was reached

In order to test the effects of temperature on the coral bioenergetic model we modified the model published by Cunning et al (2017). A diagram of the model is shown in Fig. 1a. The model tracks the changes over time of the biomass of a symbiont population *S* and its host *H*, as well as the translocation of carbon and nitrogen between the symbiotic partners. The model does not account for size dependency of the coral colony, and thus the growth rates of *S* and *H* depend on the symbiont/host ratio *S*/*H*, but not on the absolute values of *S* and *H*. This means the model describes the exponential increase or decrease of a coral colony—without accounting for density-dependent effects and growth limitations due to space or resource limitations. A more detailed analysis of the original model can be found in (Pfab et al 2022).

The parameters describing rate processes for coral and symbiont in the base model were estimated using multiple data sources from different species and environments (SI of Cunning et al (2017)). We regard them as representative of a “generic” scleractinian coral, meaning that absent further data in any particular system, they are defensible starting points for modeling. Most importantly for this paper, they have sufficient quantitative support to justify using them in this work whose primary focus is *qualitative* insight.

For most simulations in this paper we assumed that the values of parameters values in Table 2 of (Cunning et al 2017) are valid at $28^\circ \text {C}$. That implies that we recover the original model at this baseline temperature. To extend the model to account for temperature, we added two types of temperature effect to this baseline.

The first type of effect is an acceleration of metabolic processes at increased temperatures (Kooijman 2010). We assume that all rate constants are accelerated: maximum symbiont growth rate, symbiont biomass turnover rate, maximum host growth rate, host biomass turnover rate, maximum host feeding rate, maximum host DIN uptake, and maximum photosynthesis rate. We assumed that the metabolic speed factor $\alpha$ is the same for all processes, and model the metabolic speed factor by an Arrhenius function (Kooijman 2010), Fig. 2a. We chose a value of $Q_{10}=1.88$ for the temperature coefficient as this is a representative value for dinoflagellate symbionts (Anderson et al 2021) and the metabolism of corals has a similar temperature dependence (Haryanti and Hidaka 2015).

The second type of temperature effect is damage to the photosynthetic machinery. We assume that the maximum photosynthesis rate of the symbionts does not only initially increase with temperature (with the same $Q_{10}$ as the other metabolic rates), but that above a threshold this rate decreases steeply due to damage accumulation. To find a functional form for this parameter, we started with laboratory data on the photosynthetic efficiency $F_V/F_M$ from (Bellworthy and Fine 2021) and (Cunning 2024). We then assumed that the maximum photosynthesis rate in the model has a similar temperature dependence. We again calibrated the model by assuming that this rate equals at $28^\circ \text {C}$ the value given in (Cunning et al 2017). The data on $F_V/F_M$ and the maximum photosynthesis rate in our model are shown in Fig. 2b.

The “full model” includes both temperature effects (metabolic acceleration and damage to the photosynthetic machinery). We additionally analyzed two model variants that account for only one of these primary effects of temperature at a time (acceleration variant and damage variant).

The model details are described in Appendix A. A diagram that shows the model equations is shown in Fig. 7. Details for the temperature-dependent rates are given in Appendix B. The model simulations are implemented with *Wolfram Mathematica*, using the technique described in (Pfab et al 2022). The code is available online at https://github.com/ferdi-p/coral-heat-stress.

### Model simulations

#### Time course of simulations

To analyze the model, we ran simulations with different patterns of temperature over one year, while leaving all other environmental factors constant (light, DIN, and prey).

We show the directly affected rates: the metabolic speed $\alpha$ which scales all metabolic processes, and the maximal photosynthesis rate ${j_{CP}}_m$. We further show emergent properties of the system: the effective photosynthesis rate $j_{CP}$, translocation rates of carbon and nitrogen, $\rho _C$ and $\rho _N$, excess light $j_{eL}$, how much ROS is increased from the baseline $c_{ROS}-1$, symbiont expulsion $b(c_{ROS}-1)j_{ST,0}$ (which is proportional to the increase in ROS from the baseline), net and gross growth rates of the symbiont and the host, and finally the symbiont/host ratio *S*/*H*. We plotted the simulations between day 0 and 365, but started them at day $-100$ to allow the model to approach a (quasi) steady state. Initial conditions for all simulations are $H=1$, $S=0.1$, $j_{SG}=1$, and $j_{CP}=2.8$.

**Fig. 3:**
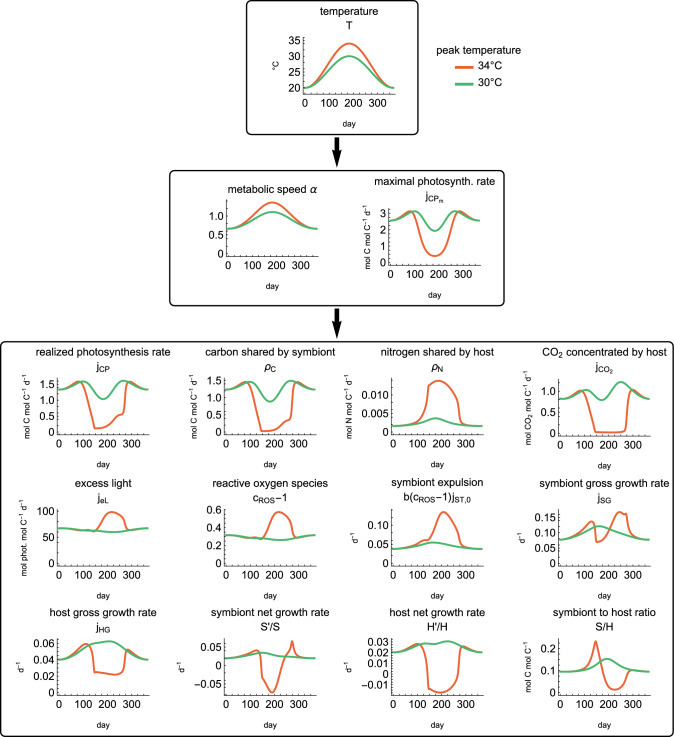
Simulations of the full model. We compare two typical seasonal patterns: a moderate summer (green) and a severe summer (red). Temperature directly affects the maximal photosynthesis rate and metabolic speed. The plots show key fluxes of the model as well as the symbiont/host ratio *S*/*H*. In the scenario with a moderate summer, the *S*/*H* ratio increases (the opposite of coral bleaching). In the scenario with a severe summer, the *S*/*H* ratio first increases but then the symbiosis breaks down and the *S*/*H* ratio drops rapidly because of symbiont expulsion. The coral bleaches. The other plotted quantities, as well as details on the bleaching cascade, are described in the main text. Environmental parameters are: light $L=30$ mol photons $\hbox {m}^{-2}$ $\hbox {d}^{-1}$, dissolved inorganic nitrogen $N=2 \times 10^{-7}$ mol N $\hbox {L}^{-1}$, and prey availability $X=2 \times 10^{-7}$ mol C $\hbox {L}^{-1}$. These values are in the range of typical values in (Cunning et al 2017)

To explore the effect of high temperatures during summer, we ran seasonal simulations of a year with a moderate and an extreme summer, Fig. 3. The seasonal temperature pattern is modeled by a sinusoidal curve. For both scenarios, temperatures start at $20^\circ \text {C}$. In the moderate summer, temperatures reach $30^\circ \text {C}$, and in the extreme summer, temperatures reach $34^\circ \text {C}$.

**Fig. 4:**
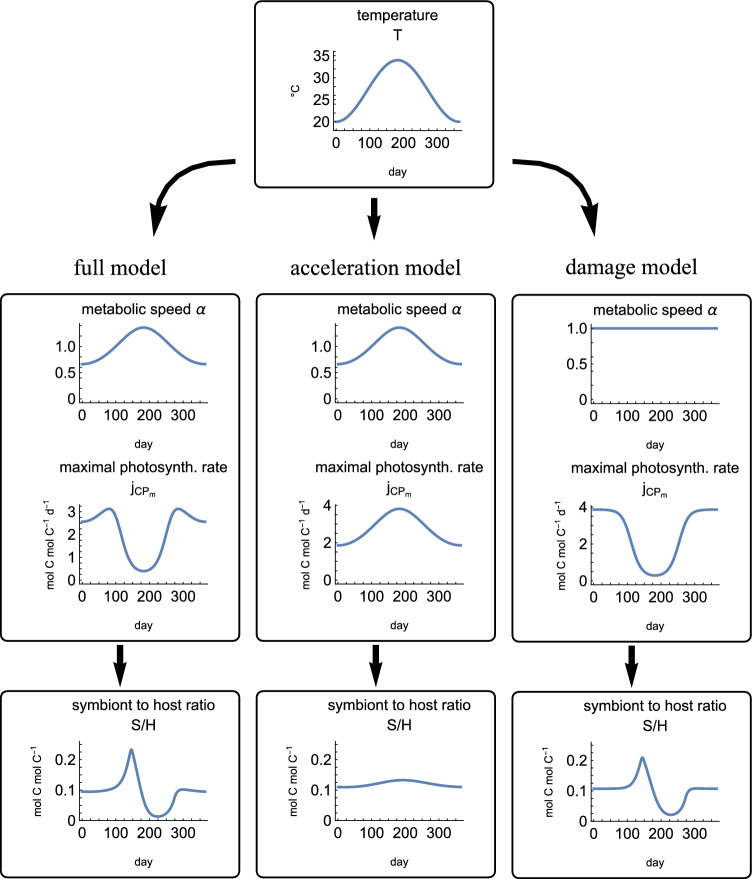
Simulations of the three model variants, only showing the symbiont/host ratio *S*/*H* as model output. With the full model and the damage model variant the *S*/*H* ratio overshoots and then crashes when temperatures are steadily increased (finally leading to coral bleaching). With the acceleration model, the *S*/*H* ratio changes only slightly—it increases during the warmer period. The full simulations are shown in Fig. S1. The parameter values are the same as in Fig. 3

Our full model has two mechanisms of temperature effects: acceleration of metabolic processes and damage to the photosynthetic machinery. To identify which of these causes the bleaching cascade, we ran further seasonal simulations to compare the full model with model variants that account only for one mechanism at a time, Fig. 4. The simulations are based on the ”extreme summer” simulation seasonal pattern described before. To keep the figure simple, we only show a selection of the model quantities (metabolic rate, maximal photosynthesis rate, and *S*/*H* ratio). More model quantities are shown in Fig. S1.

To better understand the model dynamics in seasonal environments, we repeated the seasonal simulations from Fig. 3 for a wider range of summer temperatures as well as for all model variants in Figs. S1, S2, and S3. For the first two figures, we additionally show the input fluxes into the synthesizing units for host growth, symbiont growth, and photosynthesis. In those plots, the lowest curve indicates the most limiting factor. Details are given in Appendix D.

We explored how the upkeep and breakdown of the symbiosis depend on the model assumptions by additional simulations based on the extreme summer scenario. We suspected that the $\hbox {CO}_2$ dynamics are important for the symbiosis in our model, so we ran additional simulations with unlimited $\hbox {CO}_2$ supply by setting the efficiency of the carbon concentration mechanisms (CCMs) to a very large value, $k_{CO_2}=10^6$, Fig. S4. We additionally investigated how the allocation of energy by the host to concentrate $\hbox {CO}_2$ affects the symbiosis. For this investigation, we repeated the seasonal simulations with a reduced maximal host growth rate (${j_{HG}}_m = 0.2$), which should cause the host to use less carbon for its own growth and instead allocate this toward activating CCMs ($j_{CO_2}$), Fig. S5.

We ran additional simulations in which the corals experience heat stress for 30 days to investigate whether the corals can recover after a stressful period or whether the symbiosis remains dysfunctional after the environment returns to a state that previously allowed stable symbiosis with positive growth (“hysteresis”), Fig. S6. We repeated this simulation with high and low food levels to investigate how an additional carbon source can influence the recovery of the symbiosis after heat stress. To explore how a short heat event will affect the model, we repeated the simulations with low food, but with a shorter heat shock of just 5 days, Fig. S7.

To investigate how temperature and light interact, we show time series where the temperature is gradually increased at relatively high and relatively low light levels, Fig. S8.

To explore potential intervention strategies, we ran simulations where environmental factors are altered for 42 days before and after the peak of the seasonal temperature peak, Fig. S10. The changes are a decrease of light by 20%, a decrease of dissolved inorganic nitrogen (DIN) to zero, and an increase of prey by 200%. The baseline parameter values are the same as in Fig. 3.

#### Steady states at varying temperature or DIN levels

**Fig. 5:**
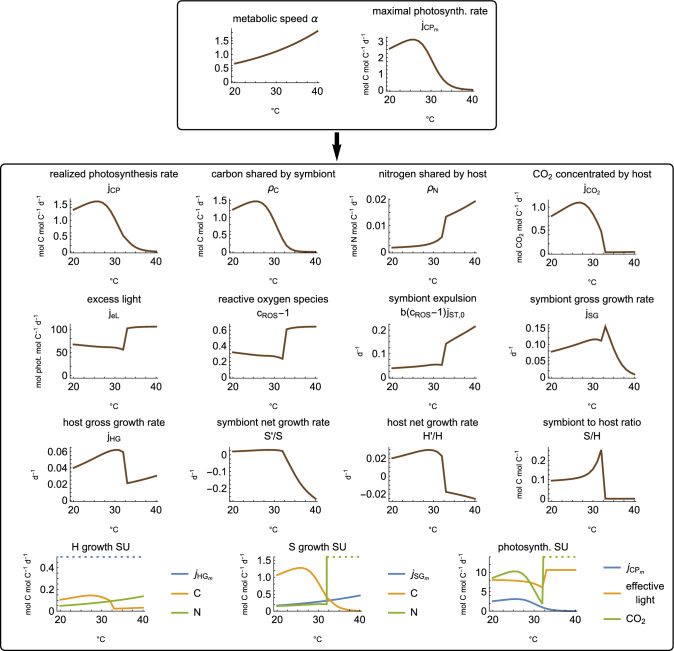
Steady state of the full model depending on temperature. The horizontal axis indicates the temperature. The plotted quantities and the parameter values are the same as in Fig. 3. Additionally, we show the inputs in the synthesizing units (SUs), described in Appendix D. Note that the steady states are relative to the symbiont and host populations—the absolute population sizes generally increase or decrease because the model does not include density dependence for the growth rate of the holobiont. Whether the population sizes increase or decrease can be seen in the plots for the net growth rates of the symbiont and the host: $S'/S$ and $H'/H$

We further studied the long-run properties of hypothetical corals for which the temperature is held constant over the course of a simulation. As known from Cunning et al (2017), the *S*/*H* ratio eventually stabilizes to a ”steady-state” value in constant environmental conditions. We ran each simulation for 1500 days and plotted the resulting model quantities as a function of the (constant) temperature, Fig. 5. The system in principle can have multiple steady states (Pfab et al 2022), but our plots show only one possible outcome for each temperature because the steady states are derived from simulations with given initial conditions. The initial conditions are the same as in Fig. 3, corresponding to a functional symbiosis. The figure in the main text only shows the full model. For the other variants, the figure is repeated in Fig. S11.

To extend our analysis for DIN, which is another environmental factor in the model, we created additional steady-state plots where we varied the DIN level, Fig. S12. For this, we fixed temperature and the other environmental factors and varied the DIN concentration between 0 and 60 $\mu$mol $\hbox {L}^{-1}$. We hypothesized a link between high DIN and the amount of energy the host uses to concentrate $\hbox {CO}_2$ for photosynthesis. To explore this connection we added simulations where we force the corals to use more of their energy for $\hbox {CO}_2$ concentration by lowering the maximal host growth rate.

#### Steady states with interacting environmental factors

**Fig. 6:**
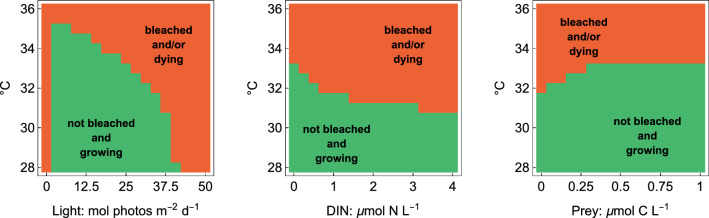
Steady state of the full model depending on temperature and either Light *L*, DIN *N*, or prey *X*. The plots indicate two regions: environments where corals are not bleached ($S/H>0.05$) and have a positive growth rate (${\rm d}H/{\rm d}t>0$), and environments where corals are bleached and/or have a negative growth rate. The environmental factors not on the axes are the same as in Fig. 3. A more detailed version of this figure with more nuanced categories for the final outputs is shown in Fig. S14

**Fig. 7:**
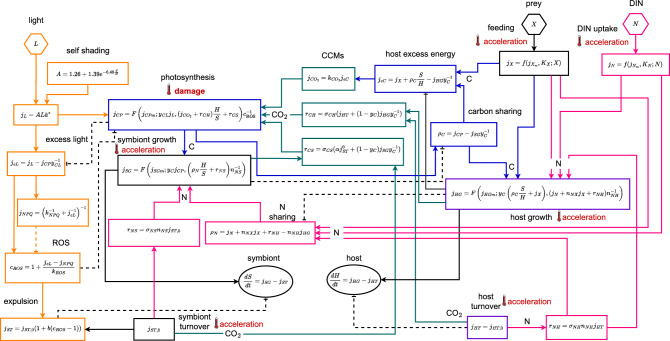
Model diagram with equations. Single substrate functional responses are defined through $f({\rm max},k;x) = {\rm max} \frac{x}{k+x}$, where *x* is the substrate, *max* is the maximal turnover speed, and *k* is the half-saturation constant. Two substrate synthesizing units are defined through $F({\rm max};x,y) = \frac{1}{\frac{1}{\rm max}+\frac{1}{x}+\frac{1}{y}-\frac{1}{x+y}}$, where *x* and *y* are the substrates and *max* is the maximal turnover speed

To further explore how temperature interacts with the other environmental factors (DIN, prey, and light), we ran long-term simulations of 1500 days over an array of different (constant) environmental conditions and summarized the overall outcome for each combination, Fig. 6. The initial conditions are the same as in the other simulations. In the plots, we categorize parameter regions by whether corals are growing and are not bleached (their symbiont/host ratio exceeds a threshold of $S/H>0.05$) - or whether corals are showing negative growth (which we interpret as dying) and/or their *S*/*H* value is lower than the threshold under which we consider the corals bleached. The figure in the main text shows only the full model. The other model variants are shown in Fig. S13. Since bleached corals can still have positive growth rates, and non-bleached corals can have negative growth rates, we aim at more nuanced insights by repeating the same plots with additional categories for the final outcomes. The final outcomes capture corals that grow and are not bleached, dying corals that are bleached, corals that grow and are bleached, and finally dying corals that are not bleached, Fig. S14.

#### Steady-state sensitivity to parameter values

We carried out a sensitivity analysis to see how sensitive the model predictions are to the parameter values, Fig. S15. For this sensitivity analysis we varied temperature and one parameter at a time, simulated the system, and extracted the final outcomes in the same fashion as for the other steady-state plots. The parameter ranges are from 0.5 to 2 times the default values from Fig. 3.

## Results and discussion

### Effects of changes in temperature on coral symbiosis

#### Temperatures below bleaching threshold

Our model simulations suggest that even moderately increased temperatures can have a range of effects on coral symbiosis. In the simulations with a moderate summer and during the initial increase of temperature in the simulations with an extreme summer (up to $32^\circ C$), the metabolic rates are accelerated and the photosystem is only slightly damaged (slightly reduced maximal photosynthetic rate ${j_{CP}}_m$), Fig. 3. As a consequence of the reduced photosynthesis, less carbon is shared with the host. This reduces the growth rate of the host, which then uses less of the nitrogen it acquires and thus *increases* nitrogen sharing. The increased nitrogen supply leads to slightly increased symbiont growth rates. The increased symbiont growth and reduced host growth together lead to an increased symbiont/host ratio during the moderate summer and at the beginning of the extreme summer (which is somewhat the opposite of coral bleaching).

Excess light, ROS, and symbiont expulsion are only marginally affected by moderately increased temperature. Excess light and ROS are slightly decreased due to increased self-shading, but symbiont expulsion is slightly increased because it is proportional to the baseline maintenance rate, which increases with temperature like the other enzymatic rates.

The steady-state plots in Fig. 5 show a similar picture: Photosynthesis first increases with temperature up to a certain threshold. After this threshold, photosynthesis decreases and the nutrient cycling is altered. The system becomes more carbon than nitrogen-limited. This increases the symbiont to host ratio and slightly decreases the net growth rates of the symbiont and the host, $S'/S$ and $H'/H$.

The model predictions at moderately increased temperatures are in accordance with experimental observations. Decreased photosynthesis is commonly observed through measures such as $F_V/F_M$ and $\hbox {O}_2$ evolution (Warner et al 1999; Gardner et al 2017b). Higher nutrient supply to the symbiont has been documented with labeled isotopes (Baker et al 2018; Rädecker et al 2021). Higher symbiont growth rates have been observed in the form of a higher mitotic index which indicates increased cell division (Strychar et al 2004). In our model, the increased nutrient supply is due to the lower nutrient consumption of the host. Only surplus nutrients are shared in our model. However, it has been proposed that symbionts may also extract nutrients from the host, which may be enhanced at increased temperatures (Morris et al 2019). Increased symbiont/host ratios at warm temperatures have been also observed in nature. Cunning and Baker (2013) report increased symbiont/host ratios before bleaching events and Sharp et al (2017); Xu et al (2017) report the highest symbiont/host ratios during summer. In contrast, other studies find the highest areal symbiont densities during winter (Fitt et al 2000; Warner et al 2002)—highlighting potential variability in responses to increased temperature (Scheufen et al 2017).

#### Temperatures above bleaching threshold

Our model suggests that extremely elevated temperatures have the opposite effect on the symbiont/host ratio. During an extreme summer, symbiosis breaks down and the coral expels its symbionts because photosynthesis is severely decreased and the production of ROS is high, Fig. 3. These model predictions align with experimental observations. Experiments show that damage and photoinhibition increase with elevated temperatures (Lesser 1997; Jones et al 1998; Lesser and Farrell 2004), that heat stress causes the photosynthetic machinery to increase ROS production, (Gardner et al 2017b; Lesser 2019), and that it results in a breakdown of the symbiosis and symbiont expulsion (Lesser 1997; Weis et al 2008).

The breakdown of the symbiosis in our model is amplified by an escalating feedback loop that involves the hosts’ carbon concentration mechanisms (CCMs). When heat stress reduces photosynthesis to a certain point, the host’s CCMs do not receive enough energy to provide sufficient $\hbox {CO}_2$ to the symbiont. This reduces photosynthesis further, eventually leading to a breakdown of photosynthesis, high ROS production, and coral bleaching. The escalating feedback between reduced photosynthesis and the reduced $\hbox {CO}_2$ supply has been proposed to be a driver in coral bleaching (Wooldridge 2009), but the magnitude of this feedback has still to be established. We discuss considerations on the photobiology and on the CCMs below in section “Rules of exchange, nutrient allocation, and carbon concentration mechanisms”.

Our model suggests a sharp temperature threshold above which corals bleach, Fig. 5. Below the threshold, the symbiosis remains functional, and the symbiont/host ratio is increased (although the growth rate of the holobiont may be reduced). Once the threshold is crossed, the symbiosis breaks down and the corals lose their symbionts. This sharp temperature response has been observed experimentally, where a few degrees Celsius determine whether corals appear healthy or lose their symbionts (Evensen et al 2021).

It is well known that not only stress intensity but also stress duration determines the severity of coral bleaching (Berkelmans 2002). This is partly reflected in our model in that the duration of heat exposure determines how many symbionts are lost during a bleaching event, Fig. S9. However, our model predicts that an arbitrarily short stress event can trigger the onset of bleaching and the system can immediately enter hysteresis—i.e., bleaching continues when the stressors is removed, Fig. S7. The reason for this is that the system enters a vicious cycle of carbon limitation further described below. The possibly unrealistic immediate onset of hysteresis is inherited from the baseline model in (Cunning et al 2017), which assumes that reserve and damage dynamics are on a fast timescale. This allows for relatively simple equations and analysis, but adding explicit dynamics for reserves and damage could add buffers against short-term environmental changes and increase the realism of the model in scenarios where the environment changes quickly (Pfab et al 2022).

Corals do not necessarily enter a permanent dysfunctional state when exposed to stress. Recovery of symbionts after a bleaching event can occur, with some examples provided in (McLachlan et al 2020). This is reflected in our seasonal simulations where the environmental conditions are such that the corals recover after the summer and the symbiont/host ratio returns to its initial value, Fig. 3. In our simulations the symbiont growth rate increases strongly during the recovery period, reflecting the decreased competition for nitrogen between symbiont individuals. Naturally, this implies that the symbionts share less of their photosynthates during recovery because they use it for their own growth processes instead. This is in agreement with experiments that demonstrate that symbionts use more of their own energy during recovery (Allen-Waller and Barott 2023).

#### Comparing the two primary temperature effects

In our model, we assumed that temperature has two types of direct effects and that the remaining model behavior is caused by downstream effects. The direct effects of increased temperature in the full model are 1) a general increase in metabolic rates and 2) damage to the photosynthetic machinery. To understand which of the two mechanisms is responsible for coral bleaching, we compared model variants with only one of those direct effects at a time. We found that damage to the photosynthetic machinery alone can cause all of the observed symptoms of heat stress, including increased symbiont/host ratios at moderately increased temperatures and coral bleaching at severely increased temperatures, Fig. 4. Metabolic acceleration alone only has marginal effects on the system, including slightly increased symbiont/host ratio and slightly increased growth rates. However, in combination with damage, metabolic acceleration decreases the bleaching threshold and makes the corals more vulnerable to temperature stress, Figs. 4 and S11. The acceleration leads to increased carbon demand by the symbiont and the host, pushing the system into the carbon-limited state where the CCMs do not provide sufficient $\hbox {CO}_2$ and the symbiosis breaks down.

### Interaction of environmental factors

Our model also demonstrates how other environmental factors interact with heat stress, Fig. 6. The interactions are in accordance with experimental observations.

High light levels have been shown experimentally to make the corals more susceptible to heat stress because excess light can overwhelm the photosynthetic machinery when it is affected by temperature stress (Lesser 1997; Huner et al 1998; Mumby et al 2001).

Similarly, increased nitrogen in the environment can enhance temperature sensitivity and reduce the bleaching threshold. In our model, additional nitrogen can push the host into the carbon-limited state where it does not have enough surplus carbon to fuel the CCMs which concentrates $\hbox {CO}_2$ for photosynthesis, Fig. S12. The role of nitrogen in facilitating coral bleaching is well documented (Donovan et al 2020; Zhao et al 2021), but other mechanisms such as raised energy demands to process nitrates and generally toxic effects of nitrogen-containing compounds could also be involved (Morris et al 2019; Donovan et al 2020).

Heterotrophic feeding of the coral can alleviate heat stress to some degree. In the model, the main reason for this effect of feeding is that it provides an additional source of carbon to fuel the CCMs and allow for photosynthesis. Particularly, feeding can allow corals to reverse hysteresis after a bleaching event and allow for recovery by restarting the CCMs and allowing for photosynthesis, Fig. S6. The positive effect of feeding on corals under stressful conditions has been observed experimentally (Tolosa et al 2011; Morris et al 2019; Huffmyer et al 2021). Besides fueling $\hbox {CO}_2$ concentration, feeding however could also increase the resilience of the symbiont due to an additional supply of nutrients such as nitrogen and phosphorus (Morris et al 2019).

Influencing those environmental factors has been proposed for interventions to protect coral reefs. Light levels could be decreased by a variety of measures, including atmospheric shading, induced turbidity, and artificial shading (National Academies of Sciences et al 2019). Feeding levels could be increased in corals in nurseries to prepare them for outplanting (Huffmyer et al 2021). Nitrogen (and other nutrients) often enters reef systems as run-off of fertilizers, and their levels on reefs could be reduced by water treatment and/or reduction of fertilizer usage (Zhao et al 2021). Simulations of these intervention scenarios confirm that reducing light, increasing feeding, and decreasing DIN can all be protective under certain conditions, Fig. S10. In the simulations with reduced light, heat stress still severely decreases the photosynthesis capacity, but the resulting excess light does not exceed the NPQ capacity and thus does not cause significant ROS production and coral bleaching. Instead of bleaching, the model predicts an increase of the symbiont/host ratio with decreased light intensity during the hottest months because the reduced photosynthesis rates decrease host growth stronger than the symbiont growth rates. In the simulations with reduced nitrogen, bleaching is delayed and reduced in its intensity because the host uses less of its energy for growth and fuels the CCMs longer during the hottest months. Similarly, in the simulations with increased host feeding, bleaching is delayed and reduced because the CCMs are fueled longer due to the extra carbon from heterotrophic feeding. Together those simulations do suggest that all of these interventions can alleviate heat stress to some degree, but the model predictions should not be interpreted quantitatively. Any quantitative specific application of the model in a specific context would need to address the model parameterization and, of particular importance, the model limitations relating nutrient dynamics and the CCMs discussed in section “Rules of exchange, nutrient allocation, and carbon concentration mechanisms”.

The model outputs also give a more nuanced insight into possible states of the symbiosis under different environmental conditions, Fig. S14. Besides the typical “non-bleached, growing” and “bleached, dying” corals, the model predicts “bleached but growing corals” when temperature and feeding are high and “not-bleached but dying” corals when temperature is high and light is low. This prediction of “bleached but growing corals” agrees with observations that corals who survive prolonged bleaching have increased feeding rates (Bessell-Browne et al 2014). “Not bleached but dying corals” are observed in the model when photosynthesis breaks down because of the heat stress, but symbiont expulsion is relatively low because of the low light. Both the symbiont and the host decline in their biomass, but at an increased symbiont/host ratio because the little carbon that is still produced by photosynthesis is used by the symbiont and not shared with the host, Fig. S8. Extremely high symbiont/host ratios however might be an artifact of the model and we are not aware of any experimental equivalence for this outcome.

### Additional considerations and future directions

Our model approach has proved to be apt in answering bulk questions on the bioenergetics of corals. The simplicity of the model allows for easy manipulation and general results without getting lost in details—or as Box (1979) famously put it: "All models are wrong but some are useful.” While simplicity is useful, it is important to be aware of the model assumptions and limitations for the interpretation of the results and for further model development.

#### Temperature effects

Temperature affects processes in corals in many ways. Our model is based on relatively simple assumptions in order to capture qualitatively how temperature affects coral symbiosis. We assume that temperature has two primary effects: a general acceleration of metabolic processes and (at higher temperatures) a decrease of the photosynthetic capacity of the symbionts. The simplicity of the assumptions allows us to draw qualitative conclusions without needing to specify many parameters that are potentially difficult to estimate. It is however necessary to consider the model assumptions carefully when interpreting the results.

**Metabolic rates** that depend on temperature in our model are the maximal symbiont and host growth rates, the symbiont and host biomass turnover rates, the maximal host feeding rate, and the maximal host DIN uptake rate. In our model, all those processes accelerate with temperature at the same rate. This assumption allows us to draw conclusions with a relatively simple model, but presumably the different processes have different temperature dependencies in reality. Adding these details might have quantitative effects on the model predictions. However qualitative effects might be limited since our simulations suggest that metabolic acceleration has little impact on the bleaching response, Fig. S3.

**Photobiology** is the other part of the model that depends on temperature. For simplicity, we assume that heat stress causes a reduction of photosynthesis without relying on any particular lower-level mechanism. We implement this by letting temperature affect one photosynthesis-related quantity directly: the maximal photosynthesis rate, which is in our model also proportional to the half-saturation constant (light level at which photosynthesis equals half of its maximal value). Initially, this quantity increases in the same way as the other metabolic parameters. At higher temperatures damage dynamics start to dominate and the maximal photosynthesis rate decreases. This approach allows us to capture the uni-modal tolerance curve, but we might miss some details. For instance, our modeling approach unifies processes that belong to the dark and the light reaction of photosynthesis, and these processes can have different temperature dependencies. Experiments suggest that damage is mostly associated with the light reaction, particularly the thylakoid membrane and the D1 protein (Warner et al 1999; Allakhverdiev et al 2008; Hoogenboom et al 2012), while effects on the dark reaction (particularly Rubisco) also include damage (Jones et al 1998) but as well an increase of the processing speed (Cen and Sage 2005). Moreover these processes are interconnected. For instance, inhibition of the light reaction can cause ROS and further damage other parts of the photosynthetic machinery (Venn et al 2008), and a slow down of the dark reaction can cause a backlog of excitation energy and additional damage to the light reaction (Jones et al 1998). These considerations suggest that granular models might give deeper insights on how temperature affects photosynthesis. However, given the complexity of the system, we believe that our simplified approach offers a reasonable base for studying how heat stress affects the coral symbiosis broadly.

#### Seasonal/diurnal cycles and nutrient reserves

Typically temperature and light intensity change seasonally. In our seasonal simulations we however only vary temperature because this is the focal environmental factor of our study. We however did run the model to steady state at different combinations of both factors—temperature, and light—and found light stress compounds heat stress, which is in accordance with experimental observations (Weis et al 2008). Adding seasonal light dynamics to our model simulations can thus be assumed to additionally contribute to coral bleaching during summer.

Besides seasonal changes, light levels obviously change during the day-night cycle. However, our model simulations assume that those day-night fluctuations can be averaged out so that it is enough to specify a daily average of light and temperature. This allows us to draw qualitative conclusions but ignores some subtleties of coral bleaching—such as ”midday expulsion” when symbionts are expelled during midday when light and temperature are highest (Wooldridge 2009). The models by Gustafsson et al (2014) and Xu et al (2022) account for daily light fluctuations in different contexts. To add daily variations to our model, the parameters might need to be adjusted (since photosynthesis saturates quickly during the day, and thus using mean light instead of daily variations could result in higher total photosynthesis). To capture the details of daily variations, it might be also more realistic to implement reserves for nitrogen, carbon, and $\hbox {CO}_2$ so that model fluxes do not hit zero during the night. Reserves have been part of the predecessors of our models by Muller et al (2009); Eynaud et al (2011), which however did not account for the host’s CCMs, which are an essential part of the bleaching cascade in the model by Cunning et al (2017). In the model by Cunning et al (2017) and our modification of it, reserves have been left out to allow for a simpler representation of the system.

#### Symbiont control, cost of symbiosis, and bleaching

In our model, the only way of directly controlling the symbiont is by expulsion, and the trigger for symbiont expulsion is the formation of ROS by the photosystem of the symbiont. ROS has been demonstrated experimentally to trigger bleaching (Lesser 1997). However, coral hosts are known to control their symbiont population by a variety of additional mechanisms. For example, it has been proposed that the host can sense carbon return from the symbiont and expels the symbiont when the return is diminished (Warner et al 1999). This possibility is reinforced by the observation that heat stress can cause bleaching without an increase of light-dependent ROS (Tolleter et al 2013). Evolutionarily, symbiont expulsion can be interpreted as a strategy to defend against harboring symbionts that cost more than they benefit (Cunning and Baker 2014; Cunning et al 2015). Proposed costs of the symbionts to the host are damaging effects of toxins such as ROS (Venn et al 2008), the energy needed to concentrate $\hbox {CO}_2$ for photosynthesis (Baker et al 2018), and the depletion of energy and nutrients (Baker et al 2018; Morris et al 2019). Our current model does not consider any direct costs of the symbiosis, but related work demonstrates how the costs of the symbiont to the host can be implemented in a similar modeling framework (Kaare-Rasmussen et al 2023).

#### Rules of exchange, nutrient allocation, and carbon concentration mechanisms

The base of the symbiosis between corals and algae is the sharing of surplus nutrients and metabolic products (e.g., photosynthate). In our model, the symbiont shares fixed carbon from photosynthesis while the host shares nitrogen and $\hbox {CO}_2$. We assume that only surplus carbon and surplus nitrogen are shared by the two players, and that $\hbox {CO}_2$ concentration by the CCMs is fueled only by excess carbon of the host.

This is a convenient modeling choice because it limits the complexity of the system and minimizes the number of parameters needed. However, interactions can be beyond the sharing of surplus. This includes symbionts extracting nutrients from the host (Morris et al 2019), the host controlling allocation of nutrients to control the symbiont density (Rädecker et al 2015), and the host stimulating the release of carbon by the symbiont through carbon release factors (Davy and Cook 2001). Also, it is likely that the assumption of how $\hbox {CO}_2$ is concentrated is a simplification of reality. In the model, the host first uses its available energy for growth and allocates only what remains for $\hbox {CO}_2$ concentration. Particularly, this strategy is not always optimal for the corals in our model. Because $\hbox {CO}_2$ is critical for photosynthesis, it can be beneficial for the corals to prioritize energy to the CCMs instead of using it immediately for growth under certain conditions, Fig. S5.

We conducted a similar analysis for the effect of nitrogen pollution and found that increased nitrogen is harmful in our model when the maximal host growth rate is relatively high such as with the default parameter values, Fig. S12. In this case, the additional nitrogen makes the host use more of its carbon for growth. In turn, insufficient energy is allocated for $\hbox {CO}_2$ concentration, and photosynthesis breaks down. However, when the maximal host growth rate is lower, additional nitrogen is generally beneficial to the system because it does not shift the system into a carbon-limited state but instead leads to a higher symbiont density that provides additional carbon to the host.

While generally decreasing the maximum host growth rate can be beneficial to keep up symbiosis in stressful conditions, more adaptive energy allocation rules could be even better strategies for the host. One possibility could be to prioritize energy allocation for a certain degree of $\hbox {CO}_2$ concentration and use the remaining energy for growth. We did some preliminary analysis and found that this can help against bleaching under stressful conditions, but we did not modify the model in this direction in order to remain close to the original model by Cunning et al (2017).

#### Parameter choices and sensitivity analysis

The model parameters are adopted from (Cunning et al 2017). They are rough estimates for the general magnitude of the modeled processes and are not specific for any species of corals or symbionts. The sensitivity analysis in Fig. S15 shows that many of the parameters can affect the bleaching threshold which defines above which temperate corals start bleaching. Parameters which (when increased) strongly decrease the bleaching threshold are the baseline symbiont maintenance rate, $j_{HT,0}^0$, the scaling factor for ROS production, $1/k_{ROS}$, the scaling parameter for the bleaching response, *b*, and the light absorbing cross section of the symbiont, $\bar{a}^*$. Parameters which (when increased) strongly decrease the bleaching threshold are the carbon efficiency of the symbiont and the host, $y_C$, the baseline maximal photosynthesis rate, $j_{CP_m}^0$, the efficiency of the CCMs, $k_{CO_2}$, and the NPQ efficiency, $k_{NPQ}$. Taken together, these highlight the importance of the symbiont properties—as would be anticipated given that heat stress most directly affects the symbiont through damage to its photosynthetic machinery.

### Conclusion

We extended a previously published coral model to include temperature effects. Increased temperature has two types of direct effects in our model: an acceleration of metabolic processes and damage to the photosynthetic machinery. Emergent features that show the integrity of the model include the following.

- In the model, moderate heat stress changes the nutrient cycling. Due to reduced photosynthesis, the symbiont shares less carbon and the host shares more nitrogen. These changes in the nutrient cycling have been reported in (Baker et al 2018; Rädecker et al 2021).
- According to the model, the altered nutrient cycling at moderate heat stress leads to decreased growth rates of the holobiont and increased symbiont/host ratios. These effects on the nutrient cycling have been reported from experiments in (Strychar et al 2004).
- Extreme heat stress leads to coral bleaching through a cascade of effects. The model predicts that due to the decreased photosynthetic capacity, captured light energy cannot be used and in turn results in the production of ROS. This has been documented by (Downs et al 2013; Gardner et al 2017b; Lesser 2019).
- The ROS trigger symbiont expulsion in the model, which also has been demonstrated experimentally (Lesser 1997; Weis et al 2008).
- The model assumes that the metabolism of the holobiont is accelerated at increased temperatures, but our simulations suggest that this acceleration has little effect on the bleaching response.
- In the model, the host’s capacity to concentrate $\hbox {CO}_2$ for photosynthesis is a main piece of stress-caused coral bleaching. The $\hbox {CO}_2$ concentration is energy-dependent, making it part of a vicious cycle in which photosynthesis breaks down during stressful conditions. While the nature and significance of this mechanism in real corals is not yet totally clear, there is evidence that it does play an important role (Jones et al 1998; Wooldridge 2009).
- The model predicts that temperature acts together with other environmental factors. Experiments agree that light stress (Lesser and Farrell 2004) and high dissolved inorganic nitrogen (Donovan et al 2020; Zhao et al 2021) can aggravate heat stress, while heterotrophic feeding of the coral can alleviate heat stress to some degree (Tolosa et al 2011; Morris et al 2019).

While these model predictions agree with observational data, we cannot be certain that the mechanisms in the model are the same as in real corals. In particular, more empirical study is needed on the role of energy-dependent $\hbox {CO}_2$ concentration and on the mechanism of heat-induced photodamage. Additional experiments, particularly covering an array of temperatures and light intensities, could be insightful and allow for more mechanistic modeling.

## Supplementary Information

Below is the link to the electronic supplementary material.

Supplementary file 1 (pdf 4184 KB)

## Appendix A: Model equations

This section describes the details of the model. The model tracks the biomass of symbiont *S* and host *H*. Additionally, it tracks various fluxes within and between the organisms, shown in Table 1. The model parameters are shown in Table 2. We fixed two obvious typos in the original formulation of the coral model, changing slightly the equations for $j_{HG}$ and $\rho _N$. A full diagram of the model equations is shown in Fig. 7.

**Table 1:** State variables and fluxes

Symbol	Description	Units
State variables		
*S*	Symbiont biomass	mol C
*H*	Host biomass	mol C
Fluxes and other auxiliary variables		
$j_X$	Prey assimilation (feeding) rate	mol C mol $\hbox {C}^{-1}$ $\hbox {d}^{-1}$
$j_N$	Nitrogen uptake rate	mol N mol $\hbox {C}^{-1}$ $\hbox {d}^{-1}$
$j_{HG}$	Host biomass formation rate	mol C mol $\hbox {C}^{-1}$ $\hbox {d}^{-1}$
$j_{HT}$	Host biomass turnover rate	mol C mol $\hbox {C}^{-1}$ $\hbox {d}^{-1}$
$r_{NH}$	Recycled nitrogen from host turnover	mol N mol $\hbox {C}^{-1}$ $\hbox {d}^{-1}$
$\rho _N$	Nitrogen shared with the symbiont	mol N mol $\hbox {C}^{-1}$ $\hbox {d}^{-1}$
$j_{eC}$	Excess carbon used to activate host CCMs	mol C mol $\hbox {C}^{-1}$ $\hbox {d}^{-1}$
$j_{CO_2}$	CO_2_ input to photosynthesis	mol $\hbox {CO}_2$ mol $\hbox {C}^{-1}$ $\hbox {d}^{-1}$
*A*	Light amplification factor	-
$j_{L}$	Light absorption rate for symbiont	mol photons mol $\hbox {C}^{-1}$ $\hbox {d}^{-1}$
$r_{CH}$	Recycled CO_2_ from host	mol $\hbox {CO}_2$ mol $\hbox {C}^{-1}$ $\hbox {d}^{-1}$
$r_{CS}$	Recycled CO_2_ from symbiont	mol $\hbox {CO}_2$ mol $\hbox {C}^{-1}$ $\hbox {d}^{-1}$
$j_{CP}$	Photosynthesis rate of symbiont	mol C mol $\hbox {C}^{-1}$ $\hbox {d}^{-1}$
$j_{eL}$	Light energy in excess of photochemistry for symbiont	mol photons mol $\hbox {C}^{-1}$ $\hbox {d}^{-1}$
$j_{NPQ}$	NPQ by the symbiont	mol photons mol $\hbox {C}^{-1}$ $\hbox {d}^{-1}$
$c_{ROS}$	ROS production by symbiont	-
$r_{NS}$	Recycled nitrogen from turnover of symbiont	mol N mol $\hbox {C}^{-1}$ $\hbox {d}^{-1}$
$j_{SG}$	Symbiont biomass formation rate	mol C mol $\hbox {C}^{-1}$ $\hbox {d}^{-1}$
$\rho _{C}$	Fixed carbon symbiont shares with host	mol C mol $\hbox {C}^{-1}$ $\hbox {d}^{-1}$
$j_{ST}$	Symbiont biomass turnover rate	mol C mol $\hbox {C}^{-1}$ $\hbox {d}^{-1}$
Temperature dependent factors		
$\alpha$	Metabolic speed factor	–
$\beta$	Damage factor	–

**Table 2:** Environmental parameters

Symbol	Description	Units
*L*	Light intensity	mol photons $\hbox {m}^{-2}$ $\hbox {d}^{-1}$
*X*	Prey availability	mol C $\hbox {L}^{-1}$
*N*	DIN concentration (dissolved inorganic nitrogen)	mol N $\hbox {L}^{-1}$

**Table 3:** Model parameters. Quantities indicated by an $\hbox {asterisk}^*$ correspond to the value at the baseline temperature $T=T_0$. The parameter values that are not temperature dependent are the same as in (Cunning et al 2017)

Symbol	Description	Units	Value
$n_{NH}$	N:C molar ratio in host biomass	mol N mol $\hbox {C}^{-1}$	0.18
$n_{NS}$	N:C molar ratio in symbiont biomass	mol N mol $\hbox {C}^{-1}$ $^{-1}$	0.13
$n_{NX}$	N:C molar ratio in prey biomass	mol N mol $\hbox {C}^{-1}$	0.2
${j^0_{HT,0}}$	Maintenance rate of host biomass at baseline temperature	mol C mol $\hbox {C}^{-1}$ $\hbox {d}^{-1}$	$0.03^*$
${j^0_{ST,0}}$	Maintenance rate of symbiont biomass at baseline temperature	mol C mol $\hbox {C}^{-1}$ $\hbox {d}^{-1}$	$0.03^*$
$\sigma _{NH}$	Proportion nitrogen turnover recycled in host	–	0.9
$\sigma _{CH}$	Proportion host metabolic CO_2_ recycled to photosynthesis	–	0.1
$\sigma _{NS}$	Proportion nitrogen turnover recycled in symbiont	–	0.9
$\sigma _{CS}$	Proportion symbiont metabolic CO_2_ recycled to photosynthesis	–	0.9
$j^0_{X_m}$	Maximum prey assimilation rate from host feeding at baseline temperature	mol C mol $\hbox {C}^{-1}$ $\hbox {d}^{-1}$	$0.13^*$
$K_X$	Half-saturation constant for prey assimilation	mol C $\hbox {L}^{-1}$	$10^{-6}$
$j^0_{N_m}$	Maximum host DIN uptake rate at baseline temperature	mol N mol $\hbox {C}^{-1}$ $\hbox {d}^{-1}$	0.035
$K_N$	Half-saturation constant for host DIN uptake	mol N $\hbox {L}^{-1}$	$1.5 \times 10^{-6}$
$k_{\text {CO}_2}$	Efficacy of $\hbox {CO}_2$ delivery to photosynthesis by host CCMs	mol $\hbox {CO}_2$ mol $\hbox {C}^{-1}$	10
$j^0_{HG_m}$	Maximum specific growth rate of host at baseline temperature	mol C mol $\hbox {C}^{-1}$ $\hbox {d}^{-1}$	$1^*$
$y_{CL}$	Quantum yield of photosynthesis for symbiont	mol C mol $\hbox {photons}^{-1}$	0.1
$y_C$	Yield of biomass formation from carbon	-	0.8
$\bar{a}^*$	Effective light-absorbing cross section of symbiont	$\hbox {m}^2$ mol $\hbox {C}^{-1}$	1.34
$k_{NPQ}$	NPQ capacity of symbiont	mol photons mol $\hbox {C}^{-1}$ $\hbox {d}^{-1}$	112
$k_{ROS}$	Excess photon energy that doubles ROS production in symbiont, relative to baseline levels	mol photons mol $\hbox {C}^{-1}$ $\hbox {d}^{-1}$	80
$j^0_{CP_m}$	Maximum specific photosynthesis rate of symbiont at baseline temperature	mol C mol $\hbox {C}^{-1}$ $\hbox {d}^{-1}$	$2.8^{*}$
${j^0_{SG}}_m$	Maximum specific growth rate of symbiont at baseline temperature	mol C mol $\hbox {C}^{-1}$ $\hbox {d}^{-1}$	$0.25^*$
*b*	Scaling parameter for bleaching response, symbiont	-	5
$w_0$	Baseline temperature	$^\circ \text {C}$	28
$Q_{10}$	$Q_{10}$ coefficient for temperature dependence of metabolism	-	1.88
$\mu _0$	Temperature at which ${j_{CP}}_m$ is reduced by 50% by damage	$^\circ$C	29.7
$\gamma$	Steepness of reduction of maximal photosynthesis rate	$^\circ\hbox {C}^{-1}$	0.58
$T_0$	Baseline temperature	$^\circ$C	28

As described in (Pfab et al 2022), the model is fully defined by tracking the slow state variables *S* and *H* and additionally at least two of the auxiliary variables. As in the previous work, we choose $j_{CP}$ and $j_{SG}$ as the additional state variables. The fluxes and other auxiliary variables are defined either directly or implicitly at their fast equilibrium states (for $j_{CP}$ and $j_{SG}$), by the equations summarized in Fig. 7.

## Appendix B: Implementing temperature dependence

### B.1 Speed of metabolic processes

We assume that the speed of metabolic processes depends on the temperature. We define the metabolic speed factor $\alpha$ through

B1 $$\begin{aligned} \begin{aligned} \alpha&= Q_{10}^\frac{T-T_0}{10} \end{aligned} \end{aligned}$$

where *T* is the temperature (in $^\circ$C), $Q_{10}$ is the temperature coefficient, and $T_0$ is the baseline temperature. We choose a value of $Q_{10}=1.88$ for the temperature coefficient as this is a typical value for Dinoflagellate symbionts (Anderson et al 2021), and we assume that the metabolism of the coral host has a similar temperature dependence.

We assume that the original model describes the situation at the baseline temperature and that the following rates accelerate with temperature: maximal symbiont growth rate ${j_{SG}}_m$, symbiont biomass turnover ${j_{ST}}_0$, maximal host growth rate ${j_{HG}}_m$, host biomass turnover ${j_{HT}}_0$, maximal host feeding rate ${j_X}_m$, and maximal host DIN uptake ${j_N}_m$. Each of these rates in the original model is multiplied by $\alpha$ to account for the temperature dependence

B2 $$\begin{aligned} \begin{aligned} {j_{SG}}_m&= \alpha {j^0_{SG}}_m\\ {j_{ST}}_0&= \alpha {j^0_{ST}}_0\\ {j_{HG}}_m&= \alpha {j^0_{HG}}_m\\ {j_{HT}}_0&= \alpha {j^0_{HT}}_0\\ {j_{X}}_m&= \alpha {j^0_{X}}_m\\ {j_{N}}_m&= \alpha {j^0_{N}}_m\\ \end{aligned} \end{aligned}$$

The metabolic speed factor $\alpha$ is shown in Fig. 2a.

### B.2 Damage to photosynthetic machinery

The maximal photosynthesis rate in our modification of the model is the product of the baseline value ${j^0_{CP}}_m$, the metabolic speed factor $\alpha$, and a temperature-dependent damage factor $\beta$

B3 $$\begin{aligned} \begin{aligned} {j_{CP}}_m = \alpha \beta {j^0_{CP}}_m \end{aligned} \end{aligned}$$

The metabolic speed factor $\alpha$ is the same as for the other temperature-dependent rates. For the damage function, we used a sigmoid function

B4 $$\begin{aligned} \begin{aligned} \beta _0&= \frac{e^{-\gamma (T-\mu _0)}}{1+e^{-\gamma (T-\mu _0)}} \end{aligned} \end{aligned}$$

where *T* is the temperature in $^\circ$C, $\mu _0$ is the temperature at which $\beta _0=1/2$, and $\gamma$ is the steepness of the function. Note that $\lim _{T\rightarrow -\infty } \beta _0= 1$ and $\lim _{T\rightarrow \infty } \beta _0 = 0$. We additionally scaled the damage function so that $j_{CP_m}$ equals its reference value ${j^0_{CP}}_m$ at the baseline temperature $T_0$,

B5 $$\begin{aligned} \begin{aligned} \beta&= \frac{\beta _0}{\beta _0 \vert _{T=T_0}} \end{aligned} \end{aligned}$$

We fit the curve for the maximal photosynthesis rate ${j_{CP}}_m$ to reported data for $F_V/F_M$. We shifted the temperature at which photosynthesis is reduced to lower temperatures by decreasing the fitted value for $\mu _0$ by ${4}^\circ$C because the data we used for $F_V/F_M$ were obtained from two different sources that are both likely to underestimate the effect of heat stress on typical corals. One of the sources describes particularly heat-adapted corals from the Red Sea, and the other source describes measures taken after only 7 h of increased temperature. The values for $\gamma$ and $\mu _0$ are reported in Table 3.

## Appendix C: Model variants

### C.1 Temperature only influences metabolic rates

In the model variant where the only effect of increased temperature is increased metabolic rates, we set the maximal photosynthesis rate constant

B6 $$\begin{aligned} \begin{aligned} \beta&= 1 \end{aligned} \end{aligned}$$

### C.2 Temperature only causes damage to photosynthetic machinery

In the model variant where the only effect of increased temperature is damage to the photosynthetic machinery, we set the metabolic speed factor to be constant

B7 $$\begin{aligned} \begin{aligned} \alpha&= 1 \end{aligned} \end{aligned}$$

## Appendix D: Synthesizing unit plots

To investigate which factors are limiting for coral growth, symbiont growth, and photosynthesis, we plot the scaled inputs to the respective SUs. Those plots are shown at different places, for example in the last row in Fig. 5. The scaled inputs are according to the model definition as follows.

**Table:**

H growth SU	
max growth rate	${j_{HG}}_m$
carbon C	$y_C \left( \rho _C \frac{S}{H}+ j_X\right)$
nitrogen N	$\left( j_N + n_{NX} j_X +r_{NH} \right) n_{NH}^{-1}$
S growth SU	
max growth rate	${j_{SG}}_m$
carbon C	$y_{C} j_{CP}$
nitrogen N	$\left( \rho _N \frac{H}{S} + r_{NS} \right) n_{NS}^{-1}$
photosynthesis SU	
max photosynthesis rate	${j_{CP}}_m$
available light	$y_{CL} j_L$
$\hbox {CO}_2$	$\left( j_{CO_2} + r_{CH} \right) \frac{H}{S} + r_{CS}$
