# Supplementary material for: Heat stress and bleaching in corals: a bioenergetic model
Source: Coral Reefs. 2024 Oct 30;43(6):1627–45. doi: 10.1007/s00338-024-02561-1 (PMC11561010; doi:10.1007/s00338-024-02561-1)
Supplement: Supplementary file 1 — (pdf 4184 KB) [file 338_2024_2561_MOESM1_ESM.pdf]

S1 Supplementary material

Heat stress and bleaching in corals: a bioenergetic model

Ferdinand Pfab[1*], A. Raine Detmer[1], Holly V. Moeller[1], Roger M. Nisbet[1], Hollie M. Putnam[2] and Ross Cunning[3]

[1*]Department of Ecology, Evolution and Marine Biology, University of California, Santa Barbara, California, USA.
[2]Department of Biological Sciences, University of Rhode Island, Kingston, Rhode Island, USA.
[3]Daniel P. Haerther Center for Conservation and Research, John G. Shedd Aquarium, Chicago, Illinois, USA.

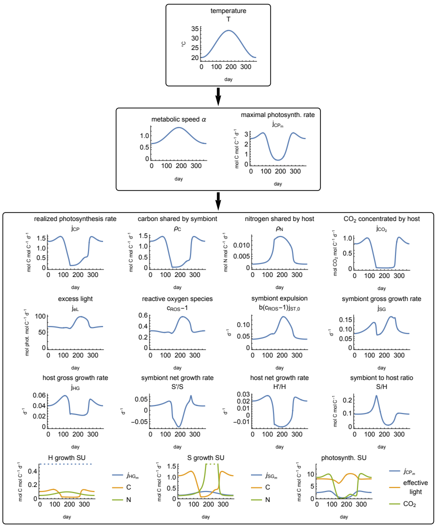

(a) Full model.

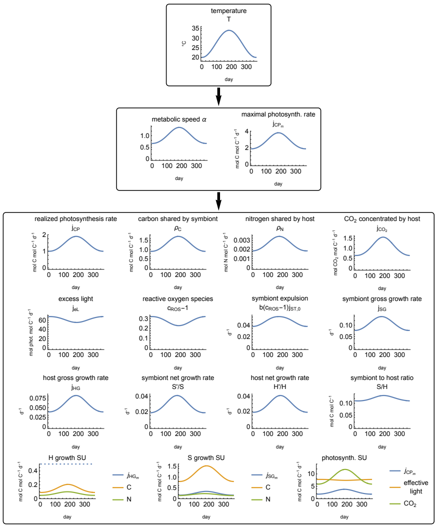

(b) Acceleration model variant.

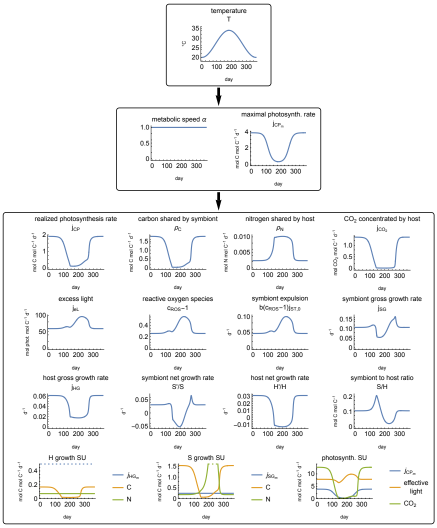

(c) Damage model variant.

Figure S1: Model simulations. Temperatures follow a typical seasonal pattern varying between 20 and 34 °C. Heat stress is extreme during the hottest part of the year, corals loose their symbionts and bleach. The plotted quantities and the parameter values are described in Fig. 3 and Fig. 5.

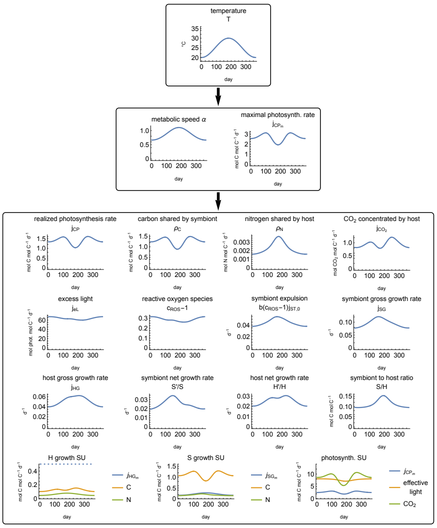

(a) Full model.

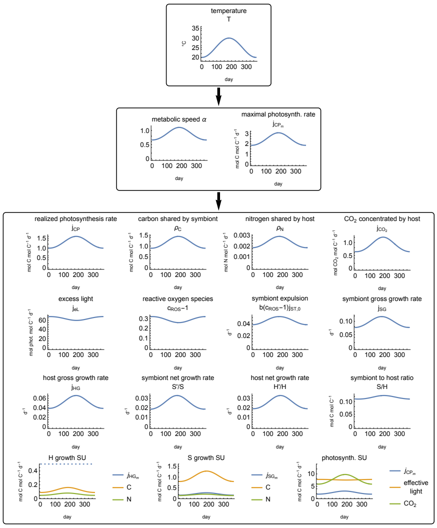

(b) Acceleration model variant.

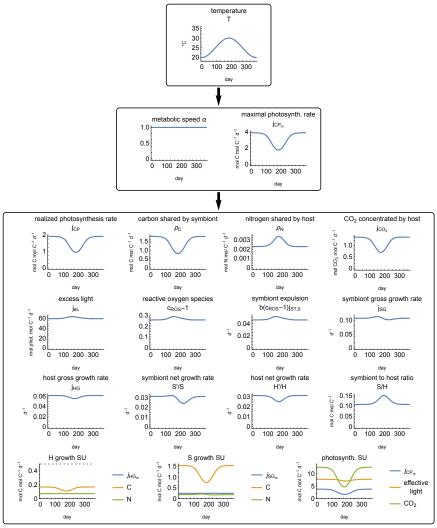

(c) Damage model variant.

Figure S2: Model simulations. Temperatures follow a typical seasonal pattern, varying between 20°C and 30°C. Temperatures in the summer are moderate and do not trigger bleaching. The symbiont-host ratio is increased during summer due to higher carbon retention by the symbionts. The plotted quantities and the parameter values are described in Fig. 3 and Fig. 5.

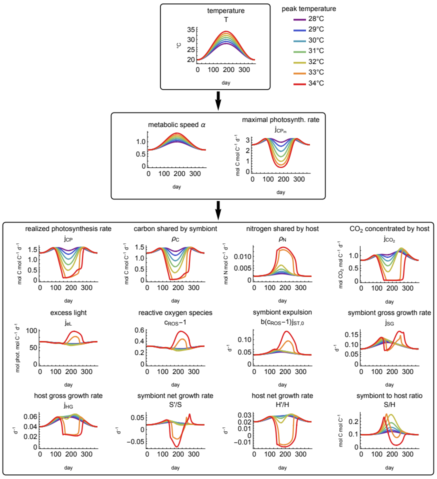

(a) Full model.

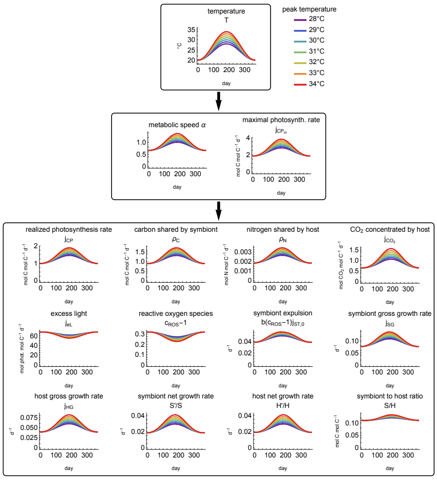

(b) Acceleration model variant.

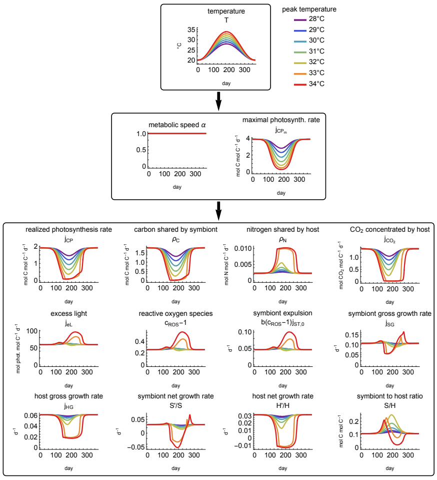

(c) Damage model variant.

Figure S3: Model simulations. Temperatures follow typical seasonal patterns with different maximal temperatures in summer. During extreme summers, corals lose their symbionts and bleach. The plotted quantities and the parameter values are described in Fig. 3.

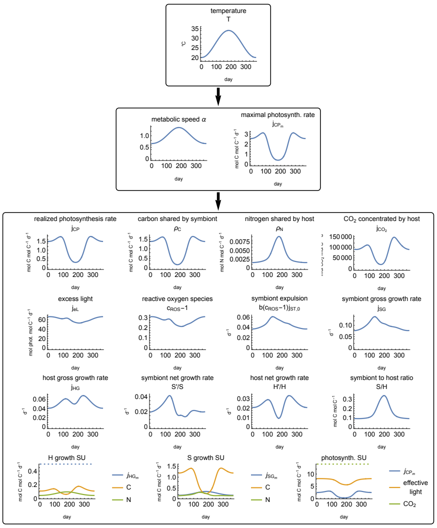

(a) Full model.

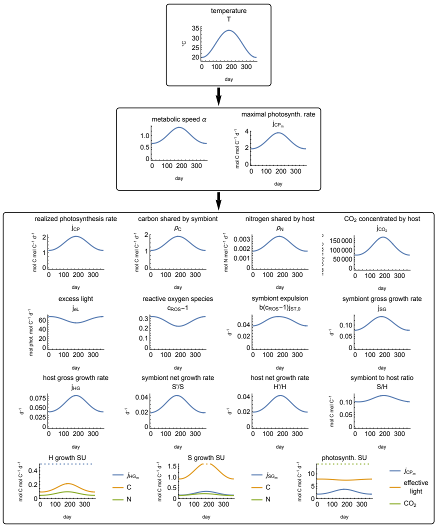

(b) Acceleration model variant.

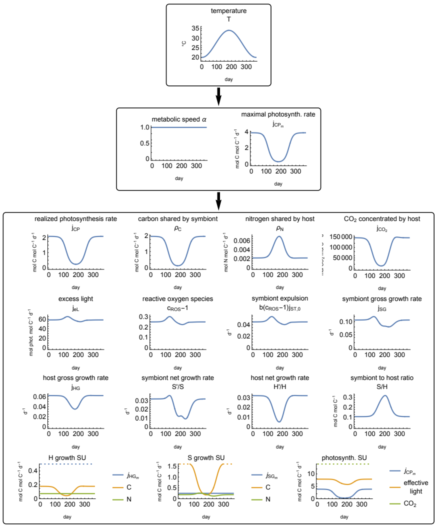

(c) Damage model variant.

Figure S4: Model simulations with unlimited CO_2. Temperatures follow a typical seasonal pattern varying between 20 and 34 °C. Heat stress is extreme during the hottest part of the year, corals lose their symbionts and bleach. The plotted quantities and the parameter values are described in Fig. 3 and Fig. 5, except that CO_2 supply for photosynthesis is unlimited by increasing the efficiency of the CCMs drastically: $k_{CO_2} = 10^6$.

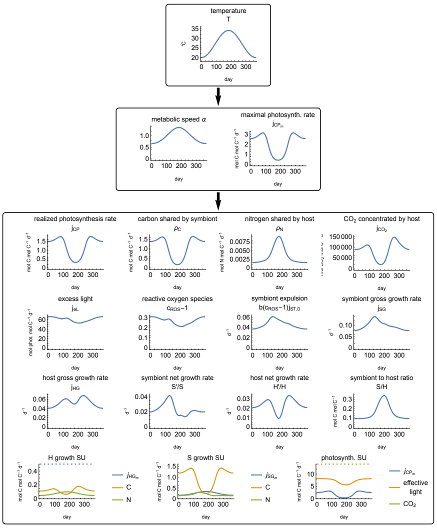

(a) Full model.

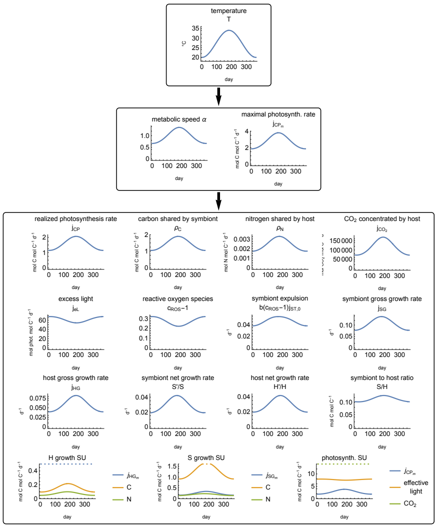

(b) Acceleration model variant.

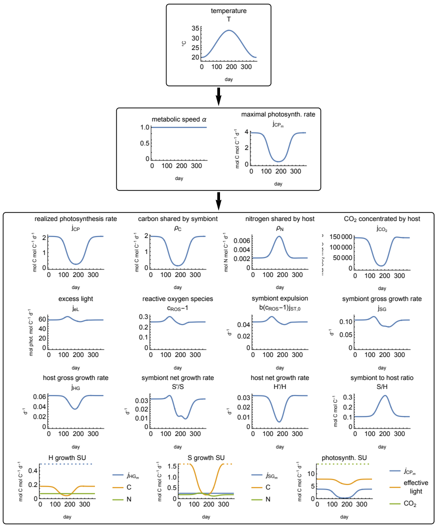

(c) Damage model variant.

Figure S5: Model simulations with lower maximal growth rate. Temperatures follow a typical seasonal pattern varying between 20 and 34 °C. Compared to the default parameters, the system grows slower in favorable conditions but bleaching is delayed and less severe. The plotted quantities and the parameter values are described in Fig. 3 and Fig. 5, except that the maximal host growth rate is lower: $j_{HG_m} = 0.2$.

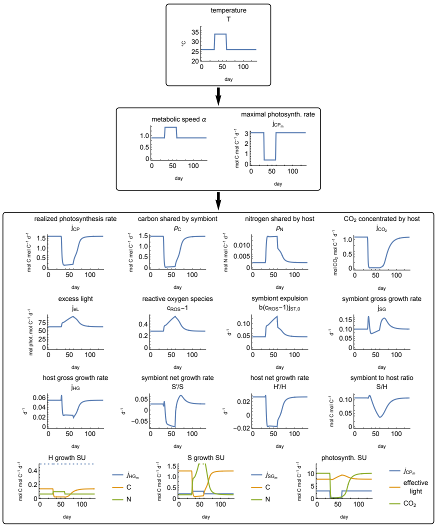

(a) The simulation shows no hysteresis: the system fully recovers after the heat shock. Parameter values as in Fig. 3.

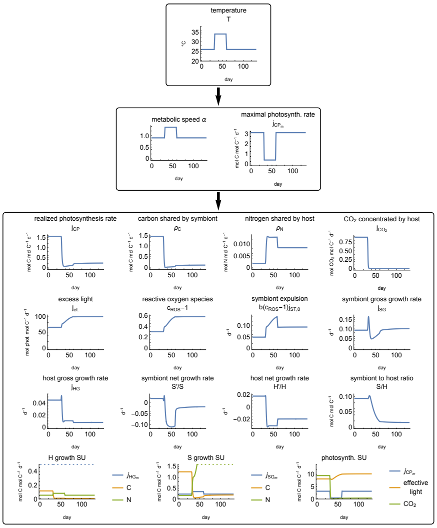

(b) Parameter values as in Fig. 3, except lower food: $X = 7.5 \times 10^{-8}$. The simulation show hysteresis: the system does not recover after the heat shock, but rather stays in an unhealthy state with a low symbiont-host ratio and negative growth rates.

Figure S6: Model simulations (full model). Temperature increases suddenly and then decreases again to its original value. When heterotrophic feeding is high enough the symbiosis is reestablished state when heat stress ends. When feeding is too low, heat stress can induce hysteresis because energy levels can get too low for the CO_2 concentration by the host and thus photosynthesis cannot be restarted. The plotted quantities are described in Fig. 3.

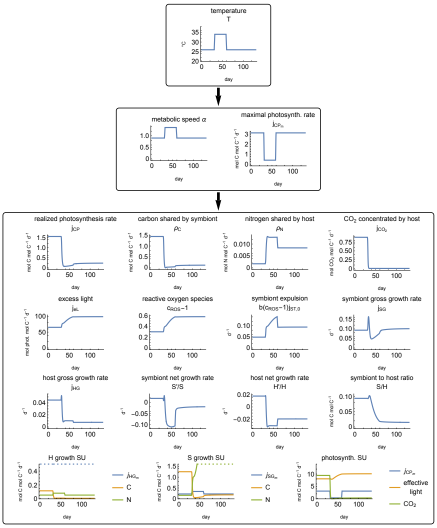

Figure S7: Model simulations (full model). Temperature increases suddenly and briefly. It then decreases again to its original value. Parameter values as in Fig. 3, except lower food: $X = 7.5 \times 10^{-8}$. The simulation show hysteresis: the system does not recover after the (very short) heat shock, but rather stays in an unhealthy state with a low symbiont-host ratio and negative growth rates. The plotted quantities are described in Fig. 3.

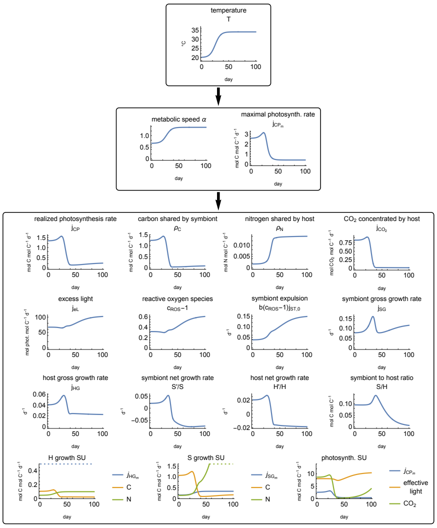

(a) Heat stress destabilizes the symbionts. The biomass of the holobiont declines and the symbiont is expelled: low S/H ratio. Parameter values as in Fig. 3.

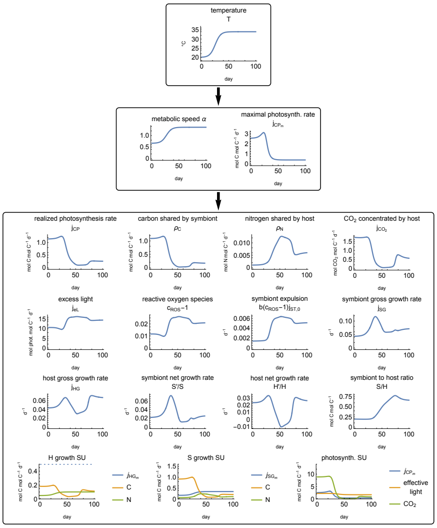

(b) Heat stress destabilizes the symbiosis. The biomass of the holobiont declines but the symbiont is not expelled: the S/H ratio increases. Parameter values as in Fig. 3, except lower light $L = 10$.

Figure S8: Model simulations (full model) with two different light levels. Temperature is increased gradually. At both light levels, heat stress leads to a breakdown of the symbiosis and a decline in the biomass of the holobiont. At high light levels, the symbiont is rapidly expelled so the system reaches a low S/H ratio. At low light levels, the symbiont is not expelled. The host biomass declines before the symbiont biomass - leading to an increased S/H ratio. The plotted quantities are described in Fig. 3 and Fig. 5.

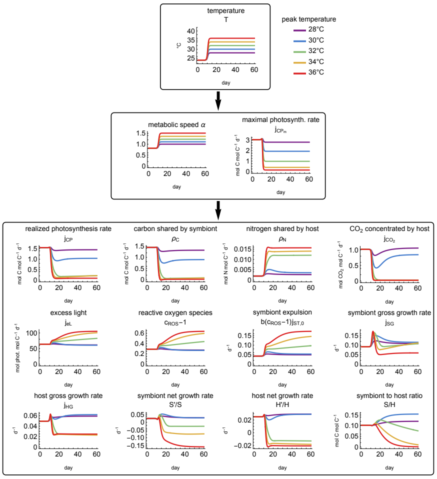

(a) Full model.

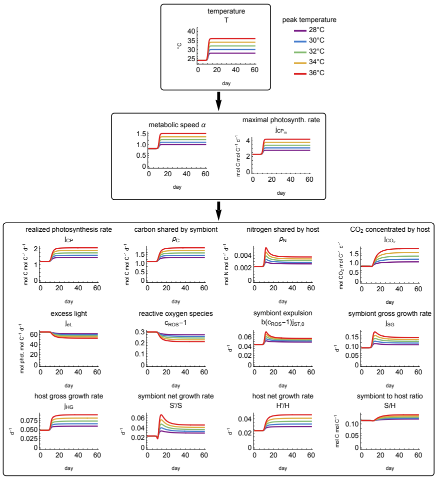

(b) Acceleration model variant.

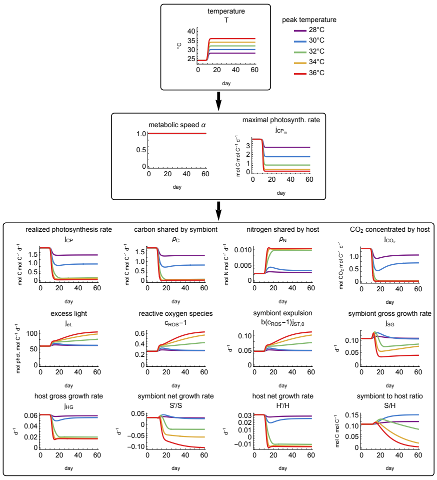

(c) Damage model variant.

Figure S9: Model simulations. Temperatures increase suddenly on day 10. Extreme temperatures lead to coral bleaching. The higher the temperature stress, the faster the symbionts are expelled. The plotted quantities and the parameter values are described in Fig. 3.

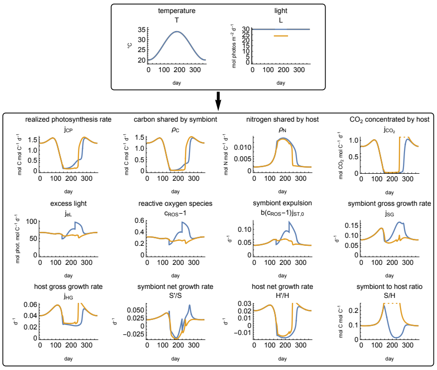

(a) Reduce light.

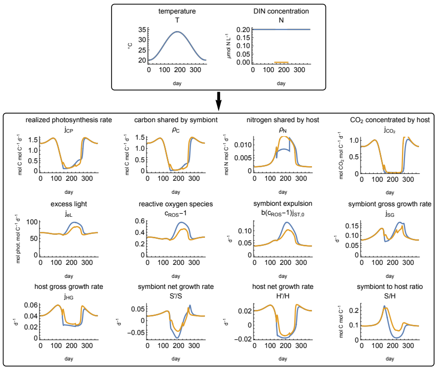

(b) Reduce DIN.

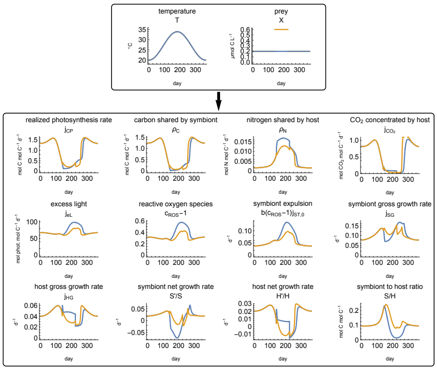

(c) Increase prey.

Figure S10: Model simulations with different intervention strategies. The blue lines correspond to a simulation without intervention. The orange lines correspond to interventions. For the interventions, we change a single environmental parameter for 42 days before and after the peak of the seasonal temperature. The changes are a decrease of light by 20%, a decrease of dissolved inorganic nitrogen (DIN) to zero, and an increase of prey by 200%. The plotted quantities and the parameter values are described in Fig. 3.

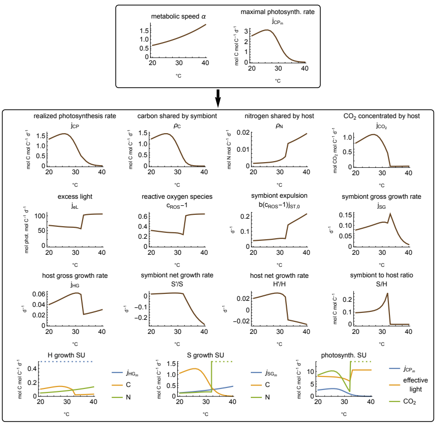

(a) Full model.

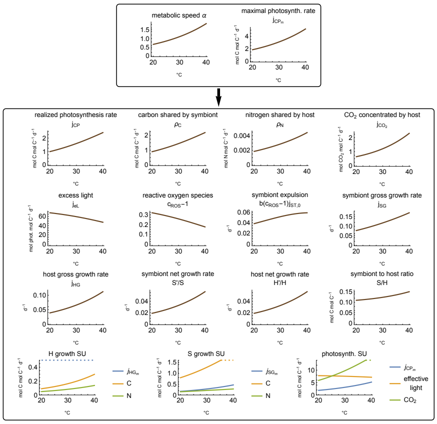

(b) Acceleration model variant.

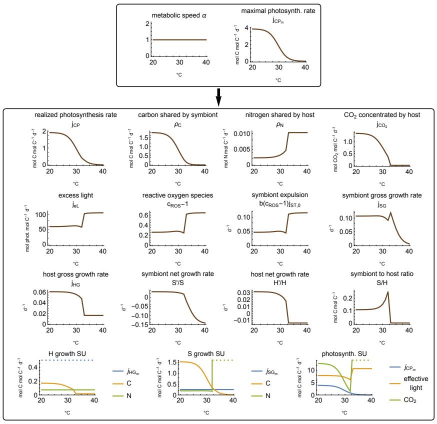

(c) Damage model variant.

Figure S11: Steady state in dependence of temperature. In full model and damage model, tipping point at critical temperature. At higher temperatures, symbiosis breaks down rapidly due to escalating feedback: photosynthesis reduced to critical level, insufficient energy for the CCMs, reduced CO_2 supply for photosynthesis, ROS production and symbiont expulsion. In the acceleration model variant, temperature does not trigger bleaching but it rather leads to increased symbiont-host ratios. In the damage model variant, increased temperature leads to coral bleaching very similarly to in the full model. The plotted quantities and the parameter values are described in Fig. 3 and Fig. 5.

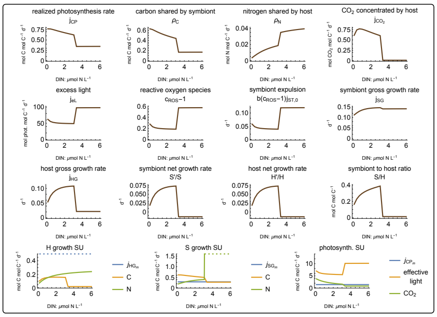

(a) Parameter values as in Fig. 3. Temperature is 31°C. The symbiosis breaks down and the corals bleach at high N levels.

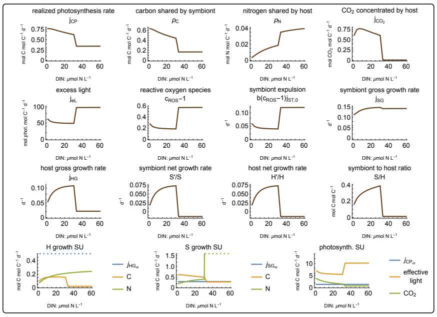

(b) Parameter values as in Fig. 3, except that the maximal host growth rate is reduced: $j_{HGm} = 0.2$. Temperature is 31°C. The symbiosis remains stable at high N levels.

Figure S12: Steady state as a function of the environmental concentration of dissolved inorganic nitrogen N (full model). High N is leading to breakdown of symbiosis only when the maximal host growth rate is high (default parameter). Otherwise increased N is not harmful because even with high N, the host does not use all C for growth; it always leaves energy for the CCMs.

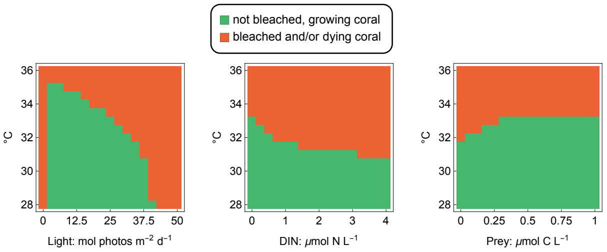

(a) Full model

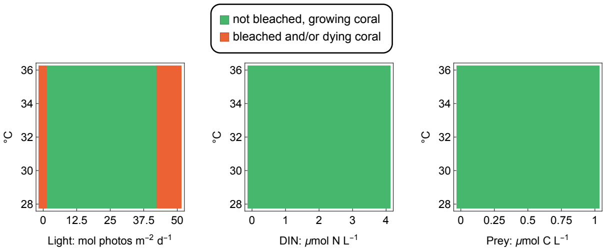

(b) Acceleration model variant

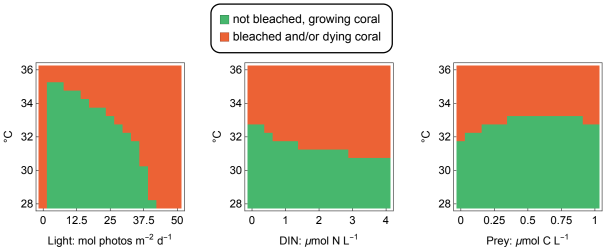

(c) Damage model variant

Figure S13: Steady state in dependence of environmental factors. Details are specified in Fig. 6.

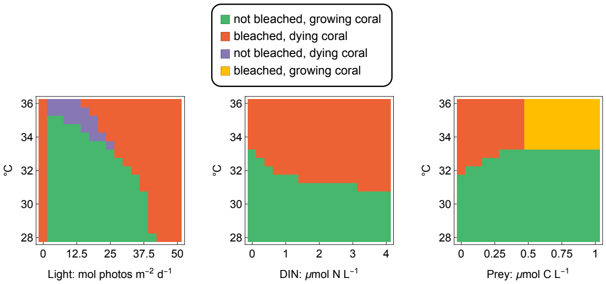

(a) Full model

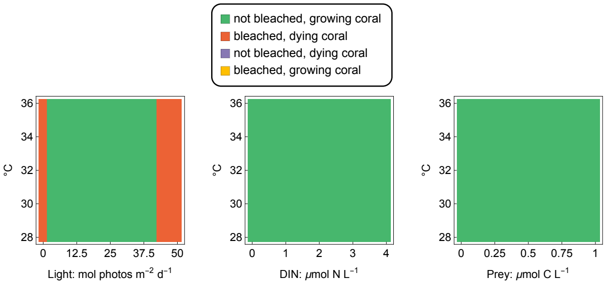

(b) Acceleration model variant

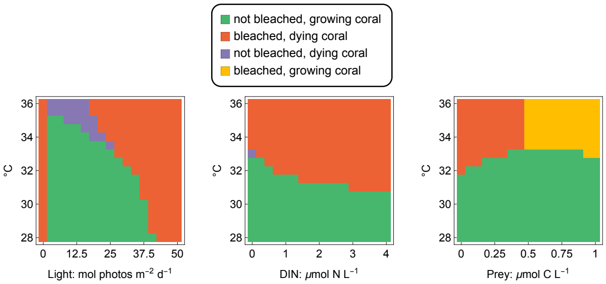

(c) Damage model variant

Figure S14: Steady state in dependence of environmental factors, indicating four distinct outcomes. Not bleached, growing coral: $S/H > 0.05$ and $dH/dt > 0$. Bleached, dying coral: $S/H \leq 0.05$ and $dH/dt \leq 0$. Not bleached, dying coral: $S/H > 0.05$ and $dH/dt \leq 0$. Bleached, growing coral: $S/H \leq 0.05$ and $dH/dt > 0$. Parameter values as in in Fig. 3.

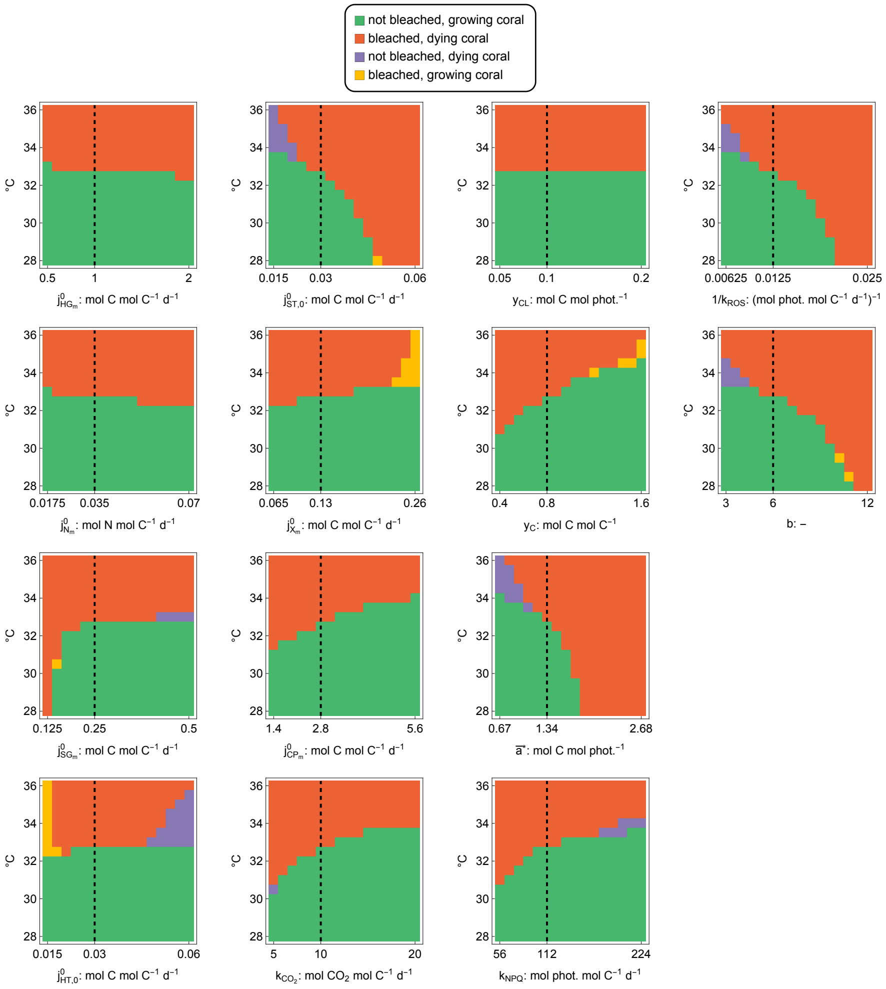

Figure S15: Sensitivity analysis showing the steady state in dependence of parameter values and temperature. The plots indicate four distinct outcomes. Not bleached, growing coral: $S/H > 0.05$ and $dH/dt > 0$. Bleached, dying coral: $S/H \leq 0.05$ and $dH/dt \leq 0$. Not bleached, dying coral: $S/H > 0.05$ and $dH/dt \leq 0$. Bleached, growing coral: $S/H \leq 0.05$ and $dH/dt > 0$. The dashed line indicates the default value of the focal parameter on the horizontal axis. Parameter values for the dashed line values and the other parameters as in Fig. 3.
